# Confidence bands in survival analysis

**DOI:** 10.1038/s41416-022-01920-5

**Published:** 2022-08-19

**Authors:** Michael C. Sachs, Adam Brand, Erin E. Gabriel

**Affiliations:** 1 Section of Biostatistics, Department of Public Health, University of Copenhagen, Copenhagen, Denmark; 2 Department of Medical Epidemiology and Biostatistics, Karolinska Institutet, Stockholm, Sweden

**Keywords:** Outcomes research, Statistics

## Abstract

**Background:**

Providing estimates of uncertainty for statistical quantities is important for statistical inference. When the statistical quantity of interest is a survival curve, which is a function over time, the appropriate type of uncertainty estimate is a confidence band constructed to account for the correlation between points on the curve, we will call this a simultaneous confidence band. This, however, is not the type of confidence band provided in standard software, which is constructed by joining the confidence intervals at given time points.

**Methods:**

We show that this type of band does not have desirable joint/simultaneous coverage properties in comparison to simultaneous bands.

**Results:**

There are different ways of constructing simultaneous confidence bands, and we find that bands based on the likelihood ratio appear to have the most desirable properties. Although there is no standard software available in the three major statistical packages to compute likelihood-based simultaneous bands, we summarise and give code to use available statistical software to construct other simultaneous forms of bands, which we illustrate using a study of colon cancer.

**Conclusions:**

There is a need for more user-friendly statistical software to compute simultaneous confidence bands using the available methods.

## Background

Ubiquitous in cancer research are survival curves, typically estimated with the Kaplan–Meier method [1], cumulative incidence curves, often estimated with the Aalen–Johansen method [2], and to a lesser extent, cumulative coefficient curves estimated from Aalen’s additive hazards model [3]. In Issue 10 of Volume 126 of the British Journal of Cancer, 4 out of 13 original articles contain estimates of survival curves. In cancer clinical trials and observational studies, curves like these are of interest because they summarise the entire distribution over the follow-up times as opposed to the binary outcome of death or progression before a particular time. This is critical in studies of deadly, late-stage cancers, since the time to progression or death is the only meaningful outcome as nearly everyone may progress or die during the follow-up period. In contrast to one-dimensional statistics like the hazard ratio, one can view the estimates of these curves as random functions of time.

In standard statistical software, along with the Kaplan–Meier estimates, one usually gets an estimate of the standard error of the survival probability estimate at each time point, and possibly the upper and lower limits of a confidence interval. It is standard practice when plotting Kaplan–Meier estimates of the survival curve to include curves that connect these pointwise confidence intervals into confidence bands; thus the confidence bands are interpreted as a visual display of the precision of the estimated curve (random function). While these confidence intervals have appropriate coverage properties when considered at a single time point, these bands do not necessarily have the correct coverage properties for the entire survival curve. The reason for this is similar to the reason why multiple testing corrections are needed when doing multiple statistical hypothesis tests. Instead, one should use some measure of the precision of the estimates of the random function that appropriately accounts for the correlation of estimates between points on the survival curve; we will call these simultaneous confidence bands. This is not to say that pointwise confidence intervals have no use. They are of course useful and appropriate for providing an interval at a particular point on the survival curve or for the cumulative incidence up to a point in follow-up, in this setting, confidence bands constructed to have 95% coverage over the full survival curve might be conservative.

**Fig. 1:**
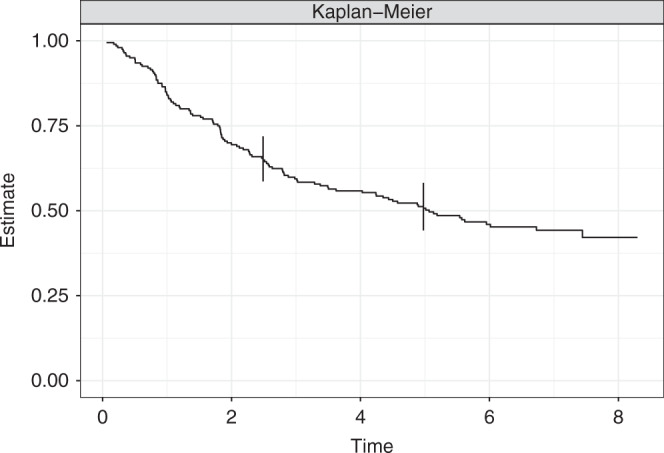
Kaplan–Meier estimates of the survival curve in the colon cancer data. The solid curve is the estimate and the vertical lines show pointwise confidence intervals at 2.5 and 5 years.

In this paper, we describe the main concepts involved in deriving simultaneous confidence bands, summarise the available methods for constructing such bands, review the statistical software one can use to estimate simultaneous confidence bands, and illustrate and compare the methods in an example. Throughout this paper, we will use as an example the data from a trial of adjuvant chemotherapy for colon cancer [4]. The study is a three-arm, randomised controlled trial comparing observation alone to Levamisole, to Levamisole plus 5-FU (a chemotherapy agent). The data are available from the survival package [5, 6] in R [7]. Figure  1 shows estimated survival curves in a random subsample of size 200 from the colon cancer data. We use a subsample of the dataset in order to make the numeric differences in the methods more apparent.

### Example: confidence intervals versus simultaneous confidence bands

In the data that produced the survival curve in Fig. 1, consider the time point of 5 years. A $1 - \alpha \%$ confidence interval for the survival probability at 5 years based on an estimate $\hat S\left( 5 \right) = 0.507$ with estimated standard error $\widehat {se}\left( 5 \right) = 0.036$ is

$$\hat S\left( 5 \right) \pm z_{1 - \alpha /2}\widehat {se}\left( 5 \right) = \left[ {0.44,\,0.58} \right]$$

where $z_{1 - \alpha /2}$ is the upper *α*⁄2 quantile of a standard normal distribution, which gives the well-known value 1.96 for *α* = 0.05. This manner of constructing confidence intervals provides the guarantee that for the year 5, the true survival will be between the lower and upper limits over ~95% of repeated samples. In our real data example in colon cancer, the pointwise confidence interval for $\hat S\left( 5 \right)$ is [0.44,0.58].

Rarely do we consider a single fixed time point when summarising distributions of times to event. Instead, estimated survival curves are plotted from some time *a* up to some time *b* which is usually the largest observed event or censoring time, where any time between these two points we can denote as *t*. If we consider the collection of pointwise confidence intervals in the range [*a, b*]

$$\{ \hat S\left( t \right) \pm z_{1 - \alpha /2}\widehat {se}\left( t \right):a \le t \le b\}$$

we have defined a *confidence band* for the survival curve over the range [*a, b*], which is the process or collection $\{ S\left( t \right):a \le t \le b\}$. We have gone from defining an interval on a one-dimensional quantity to defining a continuous banded region for a function of time. We would like a confidence band $\{ \left[ {l\left( t \right),u\left( t \right)} \right]:a \le t \le b\}$ that has simultaneous coverage properties, i.e.,

$$P\{ l\left( t \right) \le S\left( t \right) \le u\left( t \right):a \le t \le b\} \approx 1 - \alpha .$$

However, our naive construction of a pointwise confidence band does not satisfy the stronger guarantees of simultaneous coverage over the curve. The reason is that the confidence bands are for inference over multiple time points simultaneously. For instance, consider two times $t_1 \; < \; t_2$. Then

$$	P\{S(t_{1}) \in {\hat{S}}(t_{1}) \pm z_{\frac{\alpha}{2}}{\widehat{se}}(t_{1} ){{{{{\mathrm{and}}}}}}\,S(t_{2}) \in {\hat{S}}(t_{2}) \pm z_{\frac{\alpha }{2}}{\widehat{se}}( t_{2})\} \\ 	= P\left\{S(t_{1})\in {\hat{S}}(t_{1}) \pm z_{\alpha/2}{\widehat{se}}(t_{1})\right\}P\{S(t_{2}) \in {\hat{S}}(t_{2}) \pm z_{\alpha/2}{\widehat{se}}(t_{2}) \vee S(t_{1}) \\ 	\in {\hat{S}}(t_{1}) \pm z_{\alpha/2}{\widehat{se}}(t_{1})\} (1 - \alpha)P\{S(t_{2}) \in {\hat{S}}(t_{2}) \pm z_{\frac{\alpha}{2}}{\widehat{se}}(t_{2} )|S(t_{1}) \\ 	\in {\hat{S}}(t_{1}) \pm z_{\frac{\alpha}{2}}{\widehat{se}}(t_{1})\} \le 1 - \alpha$$

as $P\{ S\left( {t_2} \right) \in \hat S\left( {t_2} \right) \pm z_{\alpha /2}\widehat {se}\left( {t_2} \right) \vee S\left( {t_1} \right) \in \hat S\left( {t_1} \right) \pm z_{\alpha /2}\widehat {se}\left( {t_1} \right)\}$ is a probability, it will always be ≤1. How much less depends on exactly how dependent the estimated curves are at the two different time points. In other words, constructing a confidence band by naively connecting the points of pointwise confidence intervals is anti-conservative, because that construction does not account for the simultaneous inference. In correcting for the simultaneous inference, one should take into account the fact that the estimated survival curve at any particular time *t* depends on the history of the curve up to just before *t*.

Returning to our example dataset, and the two time points $t_1 = 2.5,t_2 = 5$, we have the pointwise confidence intervals

$$S\left( {2.5} \right) \in \left[ {0.59,0.72} \right]\;{{{{{{{\mathrm{and}}}}}}}}\;S\left( 5 \right) \in \left[ {0.44,0.58} \right]$$

whereas the simultaneous confidence bands, constructed using the methods described in the next section, are

$$S\left( {2.5} \right) \in \left[ {0.56,0.74} \right]\;{{{{{{{\mathrm{and}}}}}}}}\;S\left( 5 \right) \in \left[ {0.41,0.60} \right]$$

The limits of the simultaneous confidence bands are noticeably wider than the pointwise confidence limits, suggesting that more conservative intervals are needed to account for the simultaneous inference over multiple time points.

## Methods

We have given an example showing that pointwise confidence bands are not the same as one of the valid estimators for confidence bands, with the latter being wider. To illustrate the anti-conservativism of the pointwise confidence intervals, we must consider known survival times. Let us consider that the survival times are distributed exponentially with rate 1, so that the true survival curve is $S\left( t \right) = {{{{{{{\mathrm{exp}}}}}}}}\left( { - t} \right)$ with censoring times that are completely at random and distributed uniform over the interval [0,10]. When sampling according to this distribution and estimating pointwise 95% confidence limits over the range [0,5] it is easy to check whether the true survival curve is contained within the limits over that range. Using a sample size of 200, and running this experiment 1000 times, we find that 61.2% of the time, the true survival curve is not contained in the pointwise confidence limits at least at one point in the range [0,5]. In other words, the pointwise confidence intervals have a coverage rate of 38.8%, not 95%.

When you view a graph of an estimated survival curve with pointwise 95% confidence limits, which is common practice, how do you interpret them? It is tempting to think the graph is showing confidence limits which reasonably likely cover the true survival curve, but it is in fact quite unlikely! Simultaneous confidence bands should be used for this reason, and we now discuss ways to construct them.

### Constructing simultaneous confidence bands

Confidence intervals for the survival function at a fixed time *t* are based on a normal approximation of the sampling distribution of the Kaplan–Meier estimate at *t*. As with many one-dimensional statistics, when the Kaplan–Meier estimate is appropriately centred and scaled by its standard error, it is approximately standard normal [1]. Construction of confidence bands proceeds analogously, but using an approximation of the distribution of the entire Kaplan–Meier curve, after appropriately centring and scaling by the standard error curve. Specifically, it can be shown that

$$\sqrt n \left( {\hat S\left( t \right) - S\left( t \right)} \right) \approx - S\left( t \right)W\left( {\sigma \left( t \right)} \right)$$

as a function on the range [0, *t*], where *W*(*σ*(*t*)) is a mean zero Gaussian process with the variance function $\sigma ^2\left( t \right)$. Thus our approximation is based on a standard Gaussian process, i.e., a normal random function. It turns out that no matter what the underlying survival curve is, the scaled and centred estimated curve is well approximated by a mean zero Gaussian process, also called a Brownian motion. The properties of this type of process have been studied for a long time, and its properties well-understood. One property that is useful in constructing confidence bands is that

$$\frac{{\sqrt n }}{{\hat \sigma \left( b \right)}}\mathop {{{{{{{{{\mathrm{sup}}}}}}}}}}\limits_{s \in \left[ {a,b} \right]} \left\{ {\frac{{\left| {\hat S\left( s \right) - S\left( s \right)} \right|}}{{\hat S\left( s \right)}}} \right\} \approx \mathop {{{{{{{{{\mathrm{sup}}}}}}}}}}\limits_{0 < x < 1} \left| {W\left( x \right)} \right|$$

where $\hat \sigma ^2\left( b \right)$ is the Greenwood estimate of the variance function of $\hat S/S$ [8]. In other words, the largest absolute difference over the range [*a, b*], scaled by the estimated standard deviation at the maximum time *b* is approximately equal to the largest absolute value of a Brownian motion over the range $\left[ {0,1} \right]$. The latter follows a known distribution, and hence we can compute quantiles for it and invert this relationship to provide the desired coverage guarantee. Specifically, if we can find the value *Gγ* such that $P\{ {{{{{{{\mathrm{sup}}}}}}}}_{0 < x < 1}\left| {W\left( x \right)} \right| < G_\gamma \} \approx \gamma$, then we know that

$$P\left\{ {\frac{{\sqrt n }}{{\hat \sigma \left( b \right)}}\mathop {{{{{{{{{\mathrm{sup}}}}}}}}}}\limits_{s \in \left[ {a,b} \right]} \left\{ {\frac{{\left| {\hat S\left( s \right) - S\left( s \right)} \right|}}{{\hat S\left( s \right)}}} \right\} \; < \; G_\gamma } \right\} \approx \gamma$$

and for all *s* ∈ [*a, b*]

$$P\{ \hat S\left( s \right) - \hat S\left( s \right)\frac{{\hat \sigma \left( b \right)}}{{\sqrt n }}G_\gamma \; < \; S\left( s \right) \; < \; \hat S\left( s \right) + \hat S\left( s \right)\frac{{\hat \sigma \left( b \right)}}{{\sqrt n }}G_\gamma \} \le \gamma$$

Thus, for *γ* = 1 − *α*, $\hat S\left( s \right) \pm \hat S\left( s \right)\frac{{\hat \sigma \left( b \right)}}{{\sqrt n }}G_\gamma$ is a valid level 1−*α* confidence band for *S*(*s*) over the range [*a,b*]. These facts were discovered by Gill [9], and hence these are commonly called the “Gill bands.” Quantiles needed to calculate *G*_*γ*_ for various values of *γ* have been tabulated, so once those are found, the calculation of these bands is quite simple, as $\hat \sigma ^2\left( t \right)$ is returned by standard statistical software for survival analysis and is usually based on the Greenwood variance formula [8].

One drawback of the Gill bands is that they can be expected to be wide at early times since the variance typically is increasing as a function of time yet the construction of the bands uses $\hat \sigma ^2\left( b \right)$, the estimated variance at the maximum time, i.e., the maximum variance [10]. Hall and Wellner [11] in the same year, used a similar result to derive what are known as the “Hall-Wellner bands,” which addresses this shortcoming of the Gill bands. Specifically, the Hall–Wellner bands are

$$\hat S\left( t \right) \pm k_{1 - \alpha }\frac{{1 + n\hat \sigma ^2\left( t \right)}}{{\sqrt n }}\hat S\left( t \right)$$

for *t* ∈ [*a, b*]. The key advantage here is that since we are not dividing by *σ*(*t*), we can use *a* = 0. The critical value $k_{1 - \alpha }$ is based on the distribution of a particular transformation of a Brownian Bridge, which is another type of standard Gaussian process with a known distribution, and it can be obtained as follows: (1) fix *b* so that it is smaller than the largest uncensored observation; (2) compute $K = n\hat \sigma ^2\left( b \right)/\left( {1 + n\hat \sigma ^2\left( b \right)} \right)$, (3) compute or look up in a table $k_{1 - \alpha }$ based on the value of *K* and *α* (see Supplementary Material).

The “Equal precision” (EP) bands, developed by Nair [12], are an attempt to improve upon the Hall–Wellner bands. The EP bands are

$$\hat S\left( t \right) \pm e_{1 - \alpha }\hat \sigma \left( t \right)\hat S\left( t \right)$$

for *t* such that $a \; < \; \frac{{n\hat \sigma ^2\left( t \right)}}{{1 - n\hat \sigma ^2\left( t \right)}} \; < \; b$ and where $e_{1 - \alpha }$ is the 1 − *α* quantile of a different transformation of a Brownian Bridge from the Hall–Wellner bands. These are called equal precision bands, because the bands are proportional to the standard deviation process, just like pointwise confidence intervals are. In fact, the only difference between pointwise confidence intervals and the EP bands is the critical value. Computation of the critical value $e_{1 - \alpha }$ is not as straightforward as the critical value from a standard normal distribution, but it has been tabulated for certain values of *α, a, b* in Table 2 of Nair [12], and an approximation is given in the Supplementary Materials.

Thomas and Grunkemeier [13] developed the idea of using the likelihood ratio statistic to create confidence intervals for the survival function and Hollander et al. [14] developed this further for confidence bands. For a given survival function *S*, and a sample of possibly right-censored event times *T*_*i*_, *I* = 1, …, n the likelihood is defined

$$L\left( S \right) = \mathop {\prod }\limits_{i:{{{{{{{\mathrm{uncensored}}}}}}}}} \{S(T_i-) - S(T_i)\} \mathop {\prod }\limits_{i:{{{{{{{\mathrm{censored}}}}}}}}} S(T_i).$$

The nonparametric likelihood ratio is then defined as

$$R\left( {p,t} \right) = \frac{{{{{{{{{\mathrm{sup}}}}}}}}\{ L\left( S \right):S\left( t \right) = p,S \in \Theta \} }}{{L\left( {\hat S} \right)}}$$

where *Θ* is the set of all possible survival functions, i.e., all monotonic nonincreasing functions on [0, ∞]. For a fixed 0 ≤ *a* < *b* < ∞, the likelihood-based confidence band is

$$B = \{ S\left( t \right): - 2{{{{{{{\mathrm{log}}}}}}}}R\left( {S\left( t \right),t} \right) \le C^2\left( t \right),t \in \left[ {a,b} \right]\}$$

where *C*(*t*) is computed based on an approximation of the limiting process of the likelihood ratio, which turns out to be another transformation of the Brownian bridge. There are several variations on the likelihood ratio-based bands, using bias correction, and also based on a transformation of the cumulative hazard. These types of bands are somewhat more challenging to compute since they require numerically solving systems of equations, however, simulation studies have shown that they tend to be narrower than the EP and HW bands while maintaining correct simultaneous coverage. Another advantage is that they inherently constrain the bands to be monotonic decreasing and to be between 0 and 1.

### Bootstrap

A recurring difficulty in the computation of confidence bands for the survival curve is the computation of the critical values. While these have been tabulated in some cases, and there are formulas for approximations, these may not be readily available. To solve this problem, Akritas [15] developed the idea of using the bootstrap [16] to estimate the critical values of the limiting distribution of the transformation of the survival function described by Hall and Wellner [11]. In particular, one can estimate Kaplan–Meier curves $\hat {S}_j^{*}\left( t \right)$ for *j* = 1,…, d bootstrap replicates by resampling the data with replacement and using the usual estimation procedure. Then compute

$$A_j^{*} = \sqrt n \mathop {{{{{{{{{\mathrm{sup}}}}}}}}}}\limits_{t \in \left[ {a,b} \right]} \left\{ {\left| {\hat S_j^{*}\left( t \right) - \hat S\left( t \right)} \right|\left( {1 - K} \right)/\hat S\left( t \right)} \right\}$$

for *j* = 1,…, d. For large $d$, the critical value of the empirical distribution of the $A_j^{*}$ can be used to find a value for $k_{1 - \alpha }$ in the above description of the HW bands. In this way, no lookup tables are needed, and any value of *α* can be used.

The same idea can be applied to the other types of confidence bands by bootstrapping the appropriate statistic. In addition to the nonparametric bootstrap, the wild bootstrap can be used in survival settings [17]. The wild bootstrap approach exploits the counting process representation of estimators of the survival function, it can be computationally efficient, and it applies to a variety of different estimands, including regression models.

### Transformations

The descriptions above are all linear confidence bands, that is, without any transformation. Better performance of confidence bands in terms of coverage proportions can be obtained by using transformations. One reason for this is that the survival probability is constrained to be between 0 and 1, but the linear confidence bands do not enforce that constraint, so theoretically, the limits of the bands could extend above 1 or below 0. With large sample sizes, this is highly unlikely, nevertheless, the transformations can be used for any sample size to improve performance. The likelihood ratio-based confidence bands are naturally restricted to be between 0 and 1, hence no transformations are needed for those.

The log-transformed bands are based on

$$\{ \hat S\left( t \right)^\theta ,\hat S\left( t \right)^{1/\theta }\}$$

where for the Hall–Wellner bands,

$$\theta = \theta _{HW} = {{{{{{{\mathrm{exp}}}}}}}}\left\{ {\frac{{k_{1 - \alpha }\left( {1 + n\hat \sigma ^2\left( t \right)} \right)}}{{\sqrt n {{{{{{{\mathrm{log}}}}}}}}\hat S\left( t \right)}}} \right\}$$

while for the EP bands,

$$\theta = \theta _{EP} = {{{{{{{\mathrm{exp}}}}}}}}\left\{ {\frac{{e_{1 - \alpha }\hat \sigma \left( t \right)}}{{{{{{{{{\mathrm{log}}}}}}}}\hat S\left( t \right)}}} \right\}$$

Another type of transformation is called the arcsine-square root transform, in which the limits of the confidence bands are

$${{{{{{{\mathrm{upper}}}}}}}} = {{{{{{{\mathrm{sin}}}}}}}}^2\left( {{{{{{{{\mathrm{min}}}}}}}}\left\{ {0,{{{{{{{\mathrm{arcsin}}}}}}}}\sqrt {\hat S\left( t \right)} - \gamma \sqrt {\frac{{\hat S\left( t \right)}}{{1 - \hat S\left( t \right)}}} } \right\}} \right)$$

$${{{{{{{\mathrm{lower}}}}}}}} = {{{{{{{\mathrm{sin}}}}}}}}^2\left( {{{{{{{{\mathrm{min}}}}}}}}\left\{ \pi /2,{{{{{{{\mathrm{arcsin}}}}}}}}\sqrt {\hat S\left( t \right)} + \gamma \sqrt {\frac{{\hat S\left( t \right)}}{{1 - \hat S\left( t \right)}}} \right\} } \right)$$

where to get Hall–Wellner bands we use

$$\gamma = \gamma _{HW} = \frac{{k_{1 - \alpha }\left( {1 + n\hat \sigma ^2\left( t \right)} \right)}}{{2\sqrt n }}$$

and to get EP bands we use

$$\gamma = \gamma _{EP} = \frac{{e_{1 - \alpha }\hat \sigma \left( t \right)}}{2}$$

### Available computational methods

In order to compute the confidence bands, it is required to obtain critical values $k_{1 - \alpha }$ or $e_{1 - \alpha }$ from the distributions based on the Brownian Bridge or Brownian motion. These are not standard distributions like the Gaussian and hence they are not readily available in the back of an introductory statistics textbook or readily computable from standard functions in statistical software. Luckily, statistical software packages have been made available to construct Hall–Wellner and Equal Precision confidence bands. Unfortunately, we could not find any publicly available software packages to compute the likelihood ratio-based nor the bootstrap-based confidence bands, though we provide an example implementation in R for our particular data analysis in the Supplementary Materials.

### R

The Hall–Wellner and EP bands are implemented in the km ci package [18], based on the log transformation or the linear transformation. The code to compute them depends on having run survfit from the survival package [6] first. Then, you choose a confidence level, the lower and upper time limits, and the method, of which “hall-wellner,” “loghall,” “epband” or “logep,” give confidence bands. The bands are returned as part of the survfit object which is modified. This package uses precomputed critical values, so the only possible options for confidence levels are 90, 95 and 99%. For example, using the colon cancer dataset from survival:


library(km.ci) sfit <- survfit(Surv(time / 365.25, status) ~ 1, data = colon) sfit.loghall <- km.ci(sfit, conf.level = .95, method = “loghall”)


The km.ci does provide a function for likelihood ratio-based computation of *pointwise* confidence intervals as described by Thomas and Grunkemeier [13]. With some modification, it is possible to use this as a starting point for computation of confidence bands by finding the confidence level corresponding to the level you would need for confidence bands. In particular, starting from Eq. (7) of Hollander et al. [14], we calculate

$$\hat A = \frac{{\sqrt n \hat \sigma \left( b \right)}}{{\hat S\left( b \right)}}$$

and then $\hat d = \hat A^2/\left( {1 + \hat A^2} \right)$, where $\hat \sigma \left( b \right)$ is the estimated standard error of the estimated survival curve ($\hat S\left( b \right)$) at *b* the upper end of the interval over which the bands are desired. Then we compute or find the value $k_\alpha$ in a table such that

$$P\{ \mathop {{{{{{{{{\mathrm{sup}}}}}}}}}}\limits_{0 \le t \le \hat d} \left| {B\left( t \right)} \right| \le k_\alpha \} = \alpha$$

where *B*(*t*) is a Brownian bridge process on [0,1]. This can be done, for example, from Eq. (2.9) or Table 1 in Hall and Wellner [11]. Then rescaling this value to

$$\hat C = k_\alpha \frac{{\left( {1 + \hat A^2} \right)}}{{\hat A}}$$

we then compute $\alpha ^{*} = P\{ \hat C^2 \; < \; W\}$ where *W* is a Chi-squared random variable with 1 degree of freedom. We then use this new $\alpha ^{*}$ to set the confidence level in km.ci with method = “grunkemeier”:

lrbands <- km.ci(sfit, tl = alim, tu = blim, conf.level = 1-new.alpha, method = “grunkemeier”)

This procedure yields the basic likelihood ratio confidence bands as described by Hollander et al. [14]. They go on to develop other variations on the likelihood ratio bands that have better properties, especially in small samples. Unfortunately, we are unaware of any implementations of these bands.

### Stata

In Stata, the stcband function has been made available as an add-on package [19]. It has the same features and options as the km.ci package available in R, but it additionally has the arcsine-square root transformation implemented. To use the command, first the data needs to be identified as survival data, and then stcband is called with the options transform, nair, tlower, and tupper. The command produces a graph by default, which can be suppressed using the nograph option. The numeric values of the bands can be obtained by specifying the genhi(varname) and genlo(varname) options.


use colon stset time,f(status) scale(365.25) stcband, transform(arcsine) nair tlower(0.1) genhi(upperlim) genlo(lowerlim)


### SAS

In SAS [20], the procedures to compute confidence bands for the survival curve are available as part of the “lifetest” procedure. The option CONFBAND can be set to EP or HW, while the BANDMIN and BANDMAX options specify the time interval. Unlike in R and Stata, SAS computes the critical values using approximation formulas so that any value of *α* can be used. Two additional transformations are available in SAS: the log-log and the logit transformations. See SAS Institute Inc. [21] for more details on the options and computations.


proc lifetest confband=HW conftype=logit; time survtime*censor(1); run;


## Results

### Comparison of approaches

**Fig. 2:**
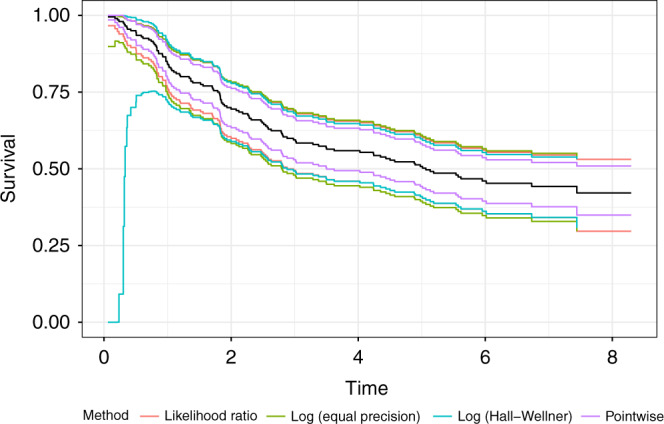
Comparison of different types of confidence bands in the colon cancer dataset. Solid black line is the estimate, and the colored lines represent the different confidence band methods.

**Fig. 3:**
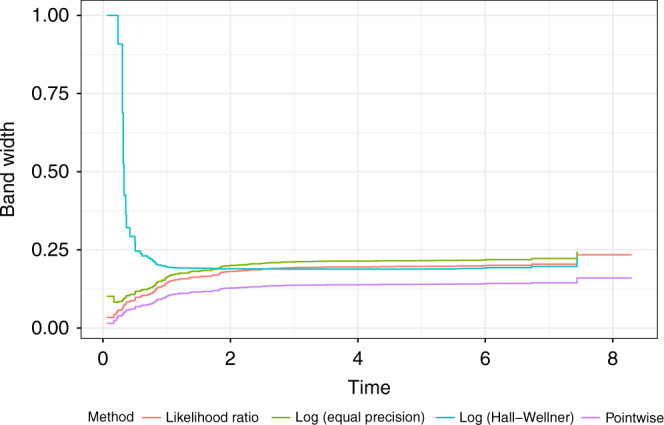
Comparison of the width of different types of confidence bands in the colon cancer dataset. Colored lines represent the different confidence band methods.

The bands described by Gill are generally thought to be inferior to the other methods, based on theoretical considerations and extensive simulation studies in Nair [12], thus we exclude them from our comparison. The different bands are shown with the survival curve estimate in Fig. 2, using a subsample of size 200 of the colon dataset to make the differences more apparent. The widths of the different bands over time are shown in Fig. 3.

The pointwise confidence intervals are the narrowest, but they do not have a nominal coverage of 95%. The log-transformed Hall–Wellner bands are wider in the earlier time ranges, but relatively narrower in the later time range. The likelihood ratio bands tend to track the log-transformed equal precision bands for earlier time ranges but then track with the Hall–Wellner. The log-transformed EP bands perform quite well in comparison to the likelihood ratio bands, and seem to be the preferred approach if computation of likelihood ratio bands is not possible. Untransformed Hall–Wellner or EP bands can potentially extend above 1 or below 0, so, if it is not feasible to use the likelihood ratio bands, it is recommended to use a transformation such as log or arcsine with equal precision bands.

## Discussion

Simultaneous confidence bands are different from pointwise confidence intervals which are often connected and plotted alongside survival curve estimates. It is inappropriate to interpret such bands as providing confidence regions for the entire curve, as they have less than nominal coverage performance (often much less).

We have described several existing methods for the construction of simultaneous confidence bands. Some of which are implemented and readily available in statistical software, and we have provided example code for using them in SAS, Stata and R. Practitioners should use these bands when plotting survival curves as is typically done in the clinical and epidemiological literature. The best performing method in simulations, the likelihood ratio approach, does not have an implementation that we can find, although we provide code for the numerical example. Implementing the general version of this approach is an area of future work for the authors.

Similar ideas can be and have been applied to derive confidence bands for other functions in survival analysis, for example, the cumulative coefficients from the additive hazards model [22], cumulative hazards, cumulative incidence curves in the competing risks setting, differences of survival curves [23], and ratios of survival curves [24]. The asymptotic theory for the Breslow estimator of the baseline hazard function in a Cox model would permit the construction of simultaneous confidence bands for the estimated survival curve at a fixed set of covariate values or “adjusted” survival curves standardised over a covariate distribution [25]. A user-friendly, general-purpose implementation for computation of such bands is warranted based on our review, as in practice, connecting the dots of pointwise intervals remains standard practice.

## Supplementary information

Confidence Bands in Survival Analysis: Supplementary Materials

Reproducibility checklist

## Data Availability

Data are publicly available from the survival package on the Comprehensive R Archive Network (CRAN).
