# Supplementary material for: Confidence bands in survival analysis
Source: Br J Cancer. 2022 Aug 19;127(9):1636–41. doi: 10.1038/s41416-022-01920-5 (PMC9596446; doi:10.1038/s41416-022-01920-5)
Supplement: Supplementary file 1 — Confidence Bands in Survival Analysis: Supplementary Materials [file 41416_2022_1920_MOESM1_ESM.pdf]

Confidence Bands in Survival Analysis: Supplementary Materials

Michael C Sachs, Adam Brand, Erin E Gabriel

The following R code reproduces and expands on the results in the main manuscript.

Background

```
library(survival)
library(km.ci)
library(broom)
library(ggplot2)


set.seed(1130)
colon <- colon[sample(1:nrow(colon), 200),]

sfit <- survfit(Surv(time / 365.25, status) ~ 1, data = colon)

tsfit <- tidy(sfit)
toplot <- rbind(data.frame(time = tsfit$time, estimate = tsfit$estimate,
                           conf.low = tsfit$conf.low,
                           conf.high = tsfit$conf.high,
                           type = "Kaplan-Meier"))

tdex <- sapply(c(2.5, 5), \(x) max(which(toplot$time <= x)))

sfitmarg <- survfit(Surv(time / 365.25, status) ~ 1, data = colon)
summary(sfitmarg, times = c(2.5, 5))
```

```
## Call: survfit(formula = Surv(time/365.25, status) ~ 1, data = colon)
##
##  time n.risk n.event survival std.err lower 95% CI upper 95% CI
##   2.5    129      70    0.649  0.0338        0.586        0.719
##   5.0     97      28    0.507  0.0355        0.442        0.582
```

```
sfitband <- km.ci(sfitmarg, method = "hall-wellner")
summary(sfitband, times = c(2.5, 5))
```

```
## Call: survfit(formula = Surv(time/365.25, status) ~ 1, data = colon)
##
##  time n.risk n.event survival std.err lower 95% CI upper 95% CI
##   2.5    129      70    0.649  0.0338        0.555        0.744
##   5.0     97      28    0.507  0.0355        0.412        0.602
```

```
ggplot(toplot, aes(x = time, y = estimate)) +
  geom_step() +
```

```
  geom_linerange(data = toplot[tdex,],
                 aes(x = time, ymin = conf.low, ymax = conf.high)) +
  facet_wrap(~type) + theme_bw() + ylim(c(0,1))
```

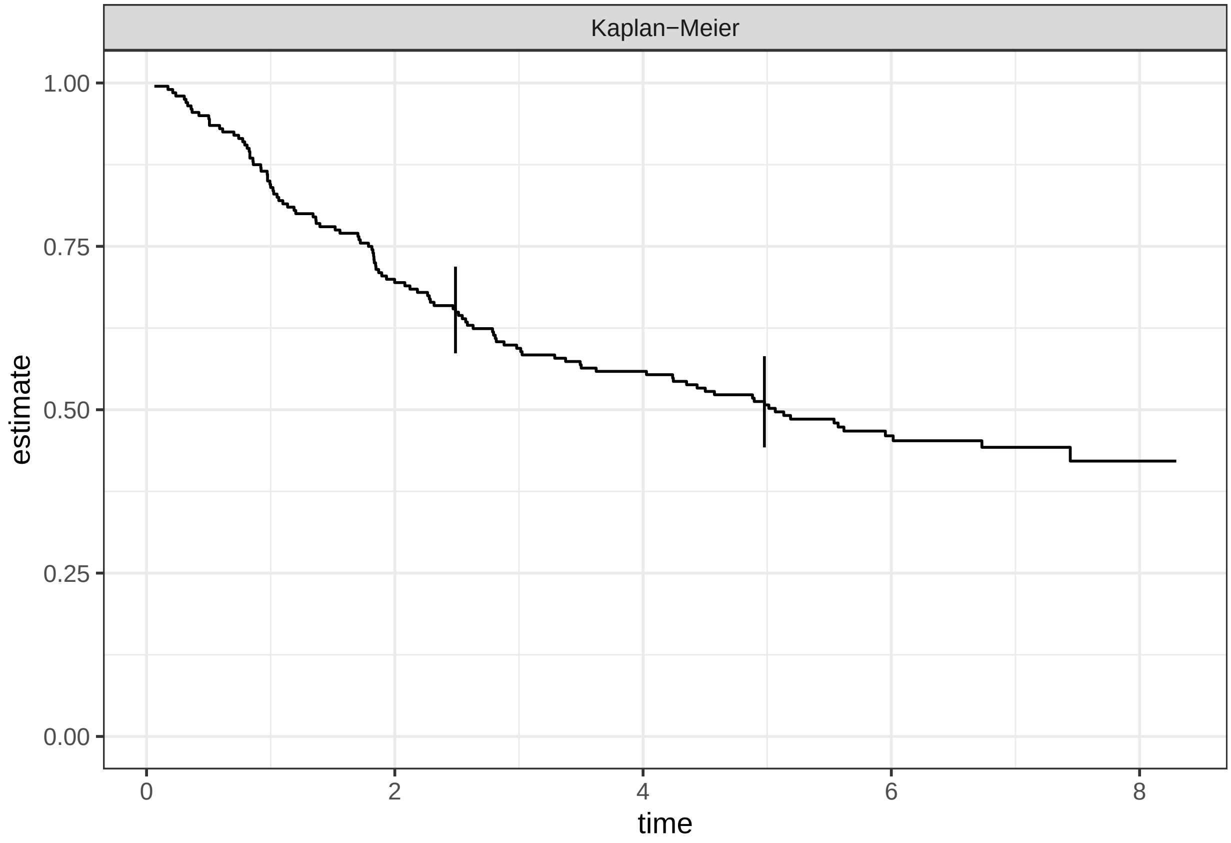


```
ggsave("Figure1.pdf")
```

```
## Saving 6.5 x 4.5 in image
```

Methods

Short simulation showing actual coverage proportions of the pointwise confidence bands.

```
viol_cov <- function(n, f1 = rexp, f2 = \(x) 1 - pexp(x) ) {
  evet <- f1(n)
  cens <- runif(n, 0, 10)

  TT <- Surv(pmin(evet, cens), 1.0* (evet <= cens))

  kmc <- survfit(TT ~ 1)
  timer <- kmc$time[kmc$time < 5]
  kmc <- summary(kmc, times = timer)
  truec <- f2(timer)

  any(truec < kmc$lower | truec > kmc$upper, na.rm = TRUE)
```

```
}

set.seed(22210)
errorrate <- mean(replicate(1000, viol_cov(200)))

errorrate
```

```
## [1] 0.612
```

Comparison of the different bands.

```
basefit <- survfit(Surv(time / 365.25, status) ~ 1, data = colon)

alim <- basefit$time[min(which(basefit$n.event > 0))]
blim <- basefit$time[max(which(basefit$n.event > 0))]
epbands <- km.ci(basefit, tl = alim, tu = blim, method = "logep")
hwbands <- km.ci(basefit, tl = alim, tu = blim, method = "loghall")
```

Likelihood ratio bands

Starting at equation 7 of Hollander et al. (1997):

```
smary <- summary(basefit)
b.dex <- max(which(smary$n.event > 0))

sigma.surv <- sqrt(smary$n) * smary$std.err[b.dex] / smary$surv[b.dex]
dhat <- sigma.surv^2 / (1 + sigma.surv^2)

Glambda <- function(a, lambda, nk= 2000) {

  k <- 1:nk
  r <- lambda * sqrt((1 - a) / a)
  d <- 1 / (1 - a)
  ksum <- (-1)^k * exp(-2 * k^2 * lambda^2) *
    (pnorm(r * (2 * k - d), lower.tail = FALSE) -
       pnorm(r * (2 * k + 2), lower.tail = FALSE))

  1 - 2 * pnorm(lambda * (a * (1 - a))^(-.5),
                lower.tail = FALSE) + 2 * sum(ksum)

}

Kalpha <- uniroot(function(x) Glambda(dhat, x) - 0.95,
                  interval = c(0,5))$root
# Kalpha approx = 1.33 From Table 1 of Hall and Wellner


C.t <- Kalpha * (1 + sigma.surv^2) / sigma.surv

# find the level alpha that gives the equivalent chisq_1 critical value
new.alpha <- pchisq(C.t^2, df = 1, lower.tail = FALSE)

lrbands <- km.ci(basefit, tl = alim, tu = blim,
                 conf.level = 1-new.alpha, method = "grunkemeier")
```

Final figures

```
figdat <- do.call(rbind, lapply(list(basefit, epbands, hwbands, lrbands),
                                function(sfit) {
  data.frame(time = sfit$time, estimate = sfit$surv,
             lower = sfit$lower, upper = sfit$upper,
             method = sfit$conf.type)
}))

figdat$method[figdat$method == "Grunkemeier"] <- "Likelihood ratio"
figdat$method[figdat$method == "log"] <- "Pointwise"

ggplot(figdat, aes(x = time, y = lower, color = method)) +
  geom_step() +
  geom_step(aes(y = upper)) +
  geom_step(data = subset(figdat, method == "Pointwise"),
            aes(y = estimate), color = "black") +
  ylab("survival") +
  theme_bw() +
  theme(legend.position = "bottom")
```

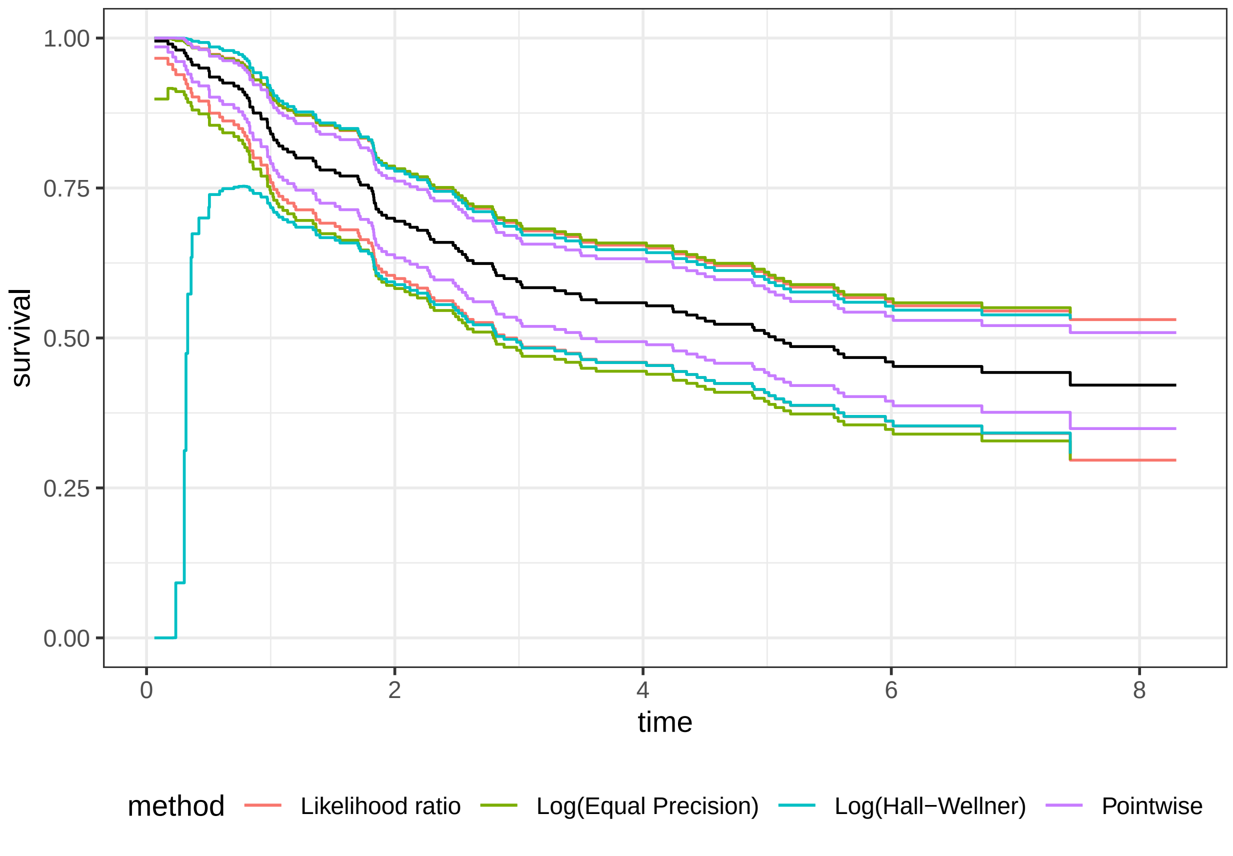


```
ggsave("Figure2.pdf")
```

```
## Saving 6.5 x 4.5 in image
```

```
figdat$width <- figdat$upper - figdat$lower

ggplot(figdat, aes(x = time, y = width, color = method)) +
  geom_step() +
  ylab("band width") +
  theme_bw() +
  theme(legend.position = "bottom")
```

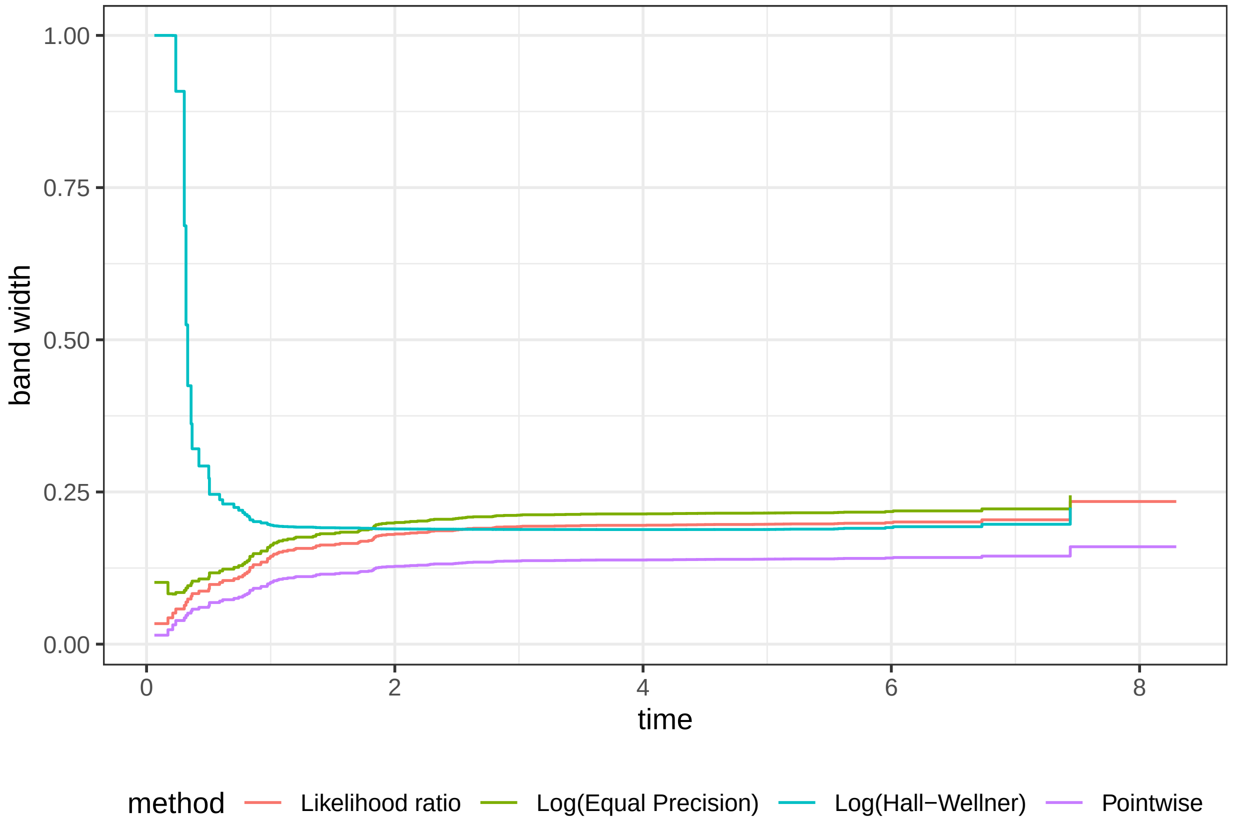


```
ggsave("Figure3.pdf")
```

```
## Saving 6.5 x 4.5 in image
```

Tables and approximations for critical values

A function for approximating λ such that

$$P(\sup_{0\le t\le a} |B^0(t)| \le \lambda) = G_a(\lambda) = 1 - \alpha.$$

Reproduces Table 1 of Hall and Wellner (1980).

```
#' Percentile for the Hall Wellner bands
#'
#' @param a Fixed value determined by the time of interest
#' @param alpha Error probability
#' @param nk Number to compute the series, larger values mean
```

```r
#' better approximation
quantile.Glambda <- function(a, alpha = .05, nk= 2000) {

  k <- 1:nk
  glamb <- function(a, lambda){

    r <- lambda * sqrt((1 - a) / a)
    d <- 1 / (1 - a)
    ksum <- (-1)^k * exp(-2 * k^2 * lambda^2) *
      (pnorm(r * (2 * k - d), lower.tail = FALSE) -
         pnorm(r * (2 * k + d), lower.tail = FALSE))

    1 - 2 * pnorm(lambda * (a * (1 - a))^(-.5),
                  lower.tail = FALSE) + 2 * sum(ksum)
  }

  Kalpha <- uniroot(function(x) glamb(a, x) - (1 - alpha),
                    interval = c(0,5))$root
  Kalpha
}

atab <- c(.1, .25, .4, .5, .6, .75, .9, .99)
alphatab <- c(.01, .05, .1, .25, .5, .75)
hwtable1 <- matrix(NA, nrow = length(alphatab), ncol = length(atab))

for(i in 1:length(atab)) {
  for(j in 1:length(alphatab)){
    hwtable1[j,i] <- quantile.Glambda(atab[i], alphatab[j], nk = 1e5)
  }
}

rownames(hwtable1) <- sprintf("alpha=%.2f", alphatab)
colnames(hwtable1) <- sprintf("a=%.2f", atab)
knitr::kable(hwtable1, digits = 3)
```

	a=0.10	a=0.25	a=0.40	a=0.50	a=0.60	a=0.75	a=0.90	a=0.99
alpha=0.01	0.851	1.256	1.470	1.552	1.600	1.626	1.628	1.628
alpha=0.05	0.682	1.014	1.198	1.273	1.321	1.354	1.358	1.358
alpha=0.10	0.599	0.894	1.062	1.133	1.181	1.217	1.224	1.224
alpha=0.25	0.471	0.711	0.854	0.920	0.967	1.008	1.019	1.019
alpha=0.50	0.356	0.544	0.663	0.720	0.765	0.809	0.827	0.828
alpha=0.75	0.272	0.420	0.518	0.567	0.608	0.652	0.675	0.676

A function for approximating the critical values for the EP bands. Reproduces Table 2 of Nair (1984).

```r
quantile.EP <- function(a, b, alpha) {

  Ax <- function(a, b, ex) {
    ex * exp(-ex^2 / 2) * (log(((1 - a) * b)) - log((a * (1 - b)))) / sqrt(8 * pi)
  }
```

```
  uniroot(function(x) Ax(a,b, x) - (alpha/2), interval = c(.5,5))$root

}

atab2 <- c(.01, .05, .1)
alphatab2 <- c(.01, .05, .1, .2)
nairtable1 <- matrix(NA, nrow = 3, ncol = 4)
for(i in 1:length(atab2)) {
  for(j in 1:length(alphatab2)) {
    nairtable1[i, j] <- quantile.EP(atab2[i], 1 - atab2[i], alphatab2[j])

  }
}


colnames(nairtable1) <- sprintf("alpha=%.2f", alphatab2)
rownames(nairtable1) <- sprintf("a=1-b=%.2f", atab2)
knitr::kable(nairtable1, digits = 2)
```

	alpha=0.01	alpha=0.05	alpha=0.10	alpha=0.20
a=1-b=0.01	3.81	3.31	3.07	2.81
a=1-b=0.05	3.68	3.16	2.91	2.62
a=1-b=0.10	3.59	3.06	2.79	2.48
